# A new species of *Glenochrysa* Esben-Petersen from Australia (Neuroptera, Chrysopidae)

**DOI:** 10.3897/zookeys.541.6643

**Published:** 2015-12-01

**Authors:** Shaun L. Winterton, Ivonne J. Garzón-Orduña

**Affiliations:** 1 California State Collection of Arthropods, California Department of Food & Agriculture, 3294 Meadowview Rd. Sacramento, California, USA 95832-1148

**Keywords:** Green lacewing, Chrysopidae, taxonomy

## Abstract

A new species of the charismatic green lacewing genus *Glenochrysa* Esben-Petersen is described from northern Western Australia. *Glenochrysa minima* **sp. n.** represents one of the smallest species of the genus. A key to species of Australian *Glenochrysa* is presented.

## Introduction

**Figure 1.**
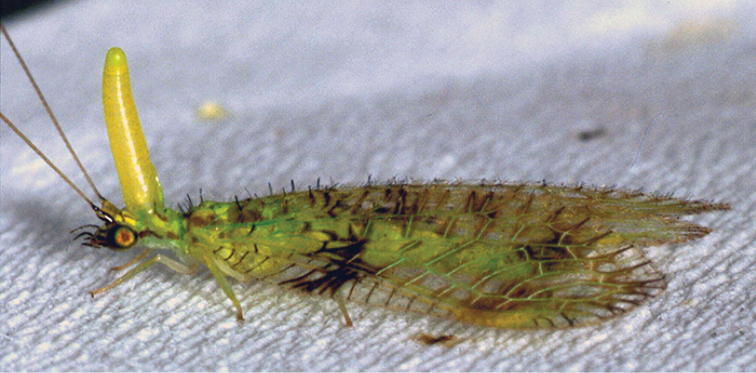
*Glenochrysa principissa* Navás, male with prothoracic ‘glenofinger’ gland everted on ceiling in Sihangwana, South Africa, (February, 2002) (from Duelli 2004).

Green lacewings (Neuroptera: Chrysopidae) are a diverse and species rich family with ca. 80 genera comprising over 1200 species in found throughout all major biogeographical regions (Brooks and Barnard 1990). The family is divided into three extant subfamilies, Apochrysinae, Nothochrysinae and Chrysopinae. The majority of the generic and species-level diversity in green lacewings is found in Chrysopinae, which includes approximately 97% of all living species. This subfamily is additionally subdivided into four tribes: Belonopterygini, Chrysopini, Leucochrysini and Ankylopterygini (Brooks and Barnard 1990; Winterton and de Freitas 2006). The most diverse tribe is Chrysopini, with over 40 genera world wide. Many genera in this tribe are nondescript green lacewings with hyaline wings requiring male genitalic dissection to confirm identity, but some are distinctive with ornate wing patterns and/or body markings. An example of this is the genus *Glenochrysa* Esben-Petersen, a genus containing 16 species distributed throughout the Afrotropical, Oriental and Australasian regions (Tjeder 1966; New 1980; Hölzel 1991; Brooks and Barnard 1990; Hölzel and Duelli 2001). A feature characteristic of this genus is the distinctively marked wings, frequently extensive, with additional iridescent embossed pustules on the wing membrane (Brooks and Barnard 1990). Other diagnostic features include in the wing, short Sc vein, meeting the costa before the wing apex, recurrent vein Cu2, and in the male terminalia, tignum and pseudopenis absent, and sternite 8+9 highly modified with medial and lateral projections bearing gonocristae (Tjeder 1966; New 1980; Brooks and Barnard 1990). A spectacular aspect of *Glenochrysa* morphology and biology is the presence of a large prothoracic gland in the male, described as the ‘glenofinger’ by Duelli (2004) (Fig. 1). While eversible prothoracic glands are known in other Chrysopidae and are used notably for defence (Güsten and Dettner 1991), the shape of this gland and use in males for courtship behaviour is possibly unique to this genus (Duelli 2004). The larva of *Glenochrysa* is a trash carrying type, confirmed in two species, *Glenochrysa ohmi* Holzel & Duelli (Hölzel and Duelli 2001) and *Glenochrysa opposita* (McLachlan) (SLW, unpublished observations).

Five previously described species of *Glenochrysa* are known from Australia (New 1980), all from the eastern state of Queensland: *Glenochrysa franzeni* Kimmins, *Glenochrysa tillyardi* New, *Glenochrysa opposita*, *Glenochrysa irregularis* (Banks) and *Glenochrysa regularis* (Banks). A sixth species of *Glenochrysa* is described here from northwestern Australia based on two male specimens. This species is atypical for the genus, due to is unusually small size, but is clearly placed in the genus based on wing and male genitalic characters. A revised key to Australian species of *Glenochrysa* is presented.

## Materials and methods

Terminology follows Tjeder (1966) and Brooks and Barnard (1990). Genitalia were macerated in 10% KOH to remove soft tissue, then rinsed in distilled water and dilute glacial acetic acid, dissected in 80% ethanol and subsequently stained with a solution of Chlorazol Black in 40% ethanol. The dissected genitalia were placed in glycerine in a genitalia vial mounted on the pin beneath the specimen.

## Taxonomy

### Glenochrysa minima sp. n.

Taxon classificationAnimaliaORDOFAMILIA

http://zoobank.org/64D03B70-AF6C-443B-AE29-E8535F8093DD

Figs 2 , 3 , 4

#### Type material.

**Holotype** male. **AUSTRALIA**: Western Australia: El Questro-Emma Gorge Resort, 15°54'16.1"S, 128°07'40.7", 20.ix.2002, Whiting, Ogden, Svensen (ANIC).

#### Paratype.

male, same data as holotype (CSCA).

#### Diagnosis.

Relatively small species (fore wing length = 7.5 mm); face without band; pronotum with lateral stripe; wing hyaline with dark venation, particularly in forewing; wings with relatively few crossveins, only two gradates in both the inner and outer gradate series, both gradate series poorly defined; arcessus straight, hooked apically.

#### Description.

**Figure 2.**
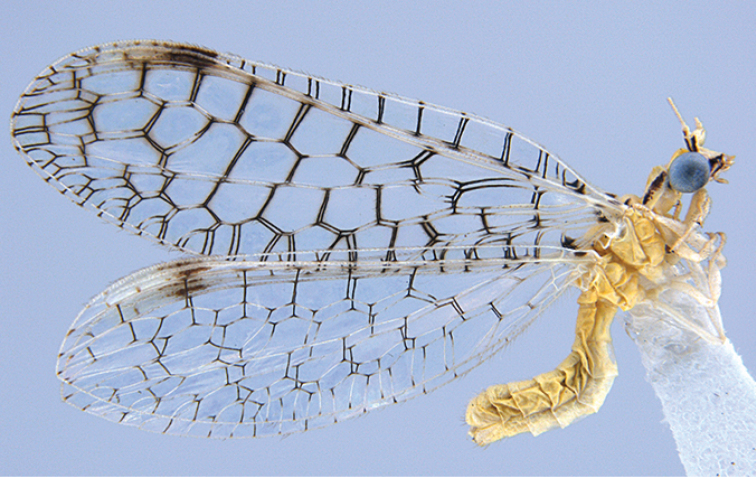
*Glenochrysa minima* sp. n., paratype male habitus. Forewing length = 7.5 mm.

**Figure 3.**
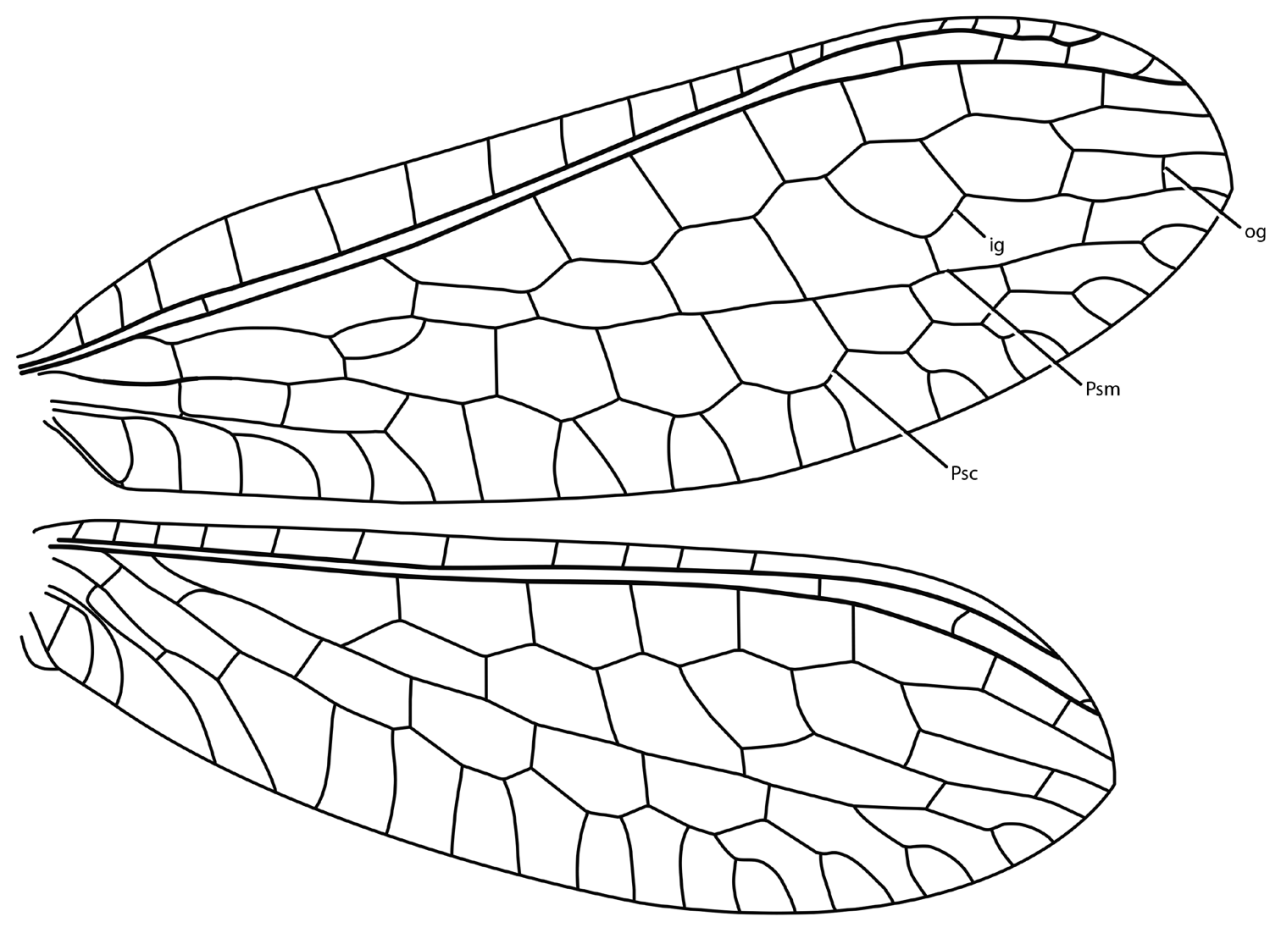
*Glenochrysa minima* sp. n., forewing (upper) and hind wing (lower). Abbreviations: Psc, pseudocubitus; Psm, pseudomedius; ig, inner gradate series; og, outer gradate series. Scale = 7.5 mm.

**Figure 4.**
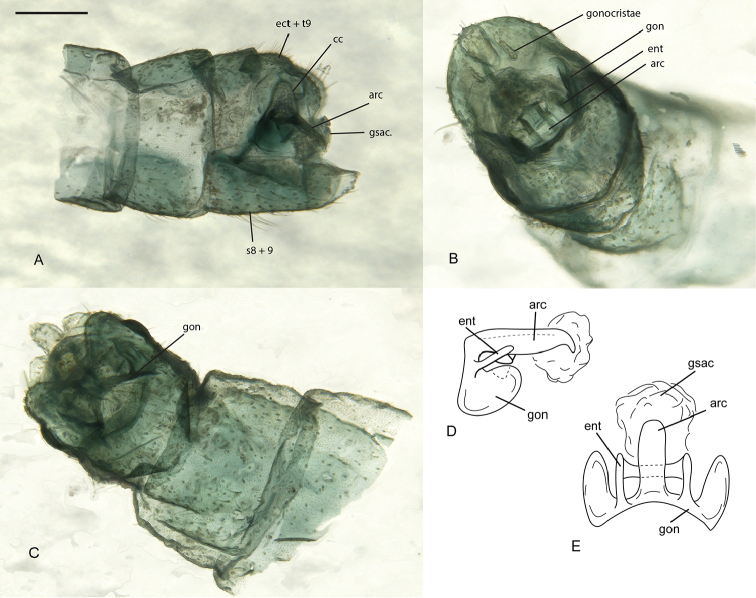
*Glenochrysa minima* sp. n., male terminalia. **A** lateral view **B** posterior view **C** dorsal view **D** gonarcus complex, lateral view **E** gonarcus complex, dorsal view. Abbreviations: ect+t9, ectoproct + tergite 9; cc, cercal callus; arc, arcessus; gsac, gonosaccus; s8+9, sternite 8+9; gon, gonarcus; ent, entoprocessus. Scale bar = 0.2 mm.

Male. Wing length (forewing: 7.5–7.8 mm, hind wing: 4.5 mm) (Figs 2–3). Overall colouration uniform green with black markings on head and thorax; wing membrane mostly unmarked. Head. Yellow, clypeus and frons paler than rest of head; vertex raised, narrow brown mark anteromedially of variable length, from less than half vertex length to full vertex length; palpi unmarked, or sometimes with dark marking laterally on distal segment; frons unmarked; gena and clypeus marked laterally with dark brown-black; small dark brown mark between eye margin and antennal base; antennal scape yellow with two lateral stripes, the anterior stripe slightly wider than the posterior stripe; pedicel yellow with small dark mark laterally; flagellum uniform yellow (broken). Thorax. Prothorax green dorsally, paler on sides, lateral margin with dark brown stripe; anterior margin of prothorax slightly raised where the ‘glenofinger’ organ is everted; mesothorax yellow-green with dark brown diagonal marking anterolaterally on mesonotum; metathorax entirely yellow-green, unmarked; legs very pale yellow-green, unmarked, base of claw broad; forewing hyaline, longitudinal veins and costal margin largely pale, crossveins dark with narrow infuscation on membrane adjacent to crossveins, more pronounced on first costal crossvein and where vein 3A meeting wing margin; pterostigma dark proximally, pale distally; two widely spaced gradates in each series, with gradates overlapping; inner gradate series not meeting Psm; hindwing similarly hyaline with dark crossveins, although with less intensity and no shading of adjacent membrane; two inner gradates while the outer gradate series differs from one to two between wings. Abdomen. Uniform yellow-green, pale setae present on sclerites of posterior segments; mostly shorter and sparser on anterior segments than rest of abdomen. Genitalia (Fig. 4). Cercal callus with ca. 28 trichobothria and distinct apodeme anteroventrally from cercal callus; ectoproct +tergite 9 broadly rounded in lateral view, inner margin angled in dorsal view, margin with field or erect strong setae posteriorly; sternite 8+9 subquadrangular, posterior margin trilobed with median lobe apex rounded, projecting beyond lateral lobes, lateral lobes with gonocristae along interior margins; gonapsis relatively small; gonarcus arched, arms rounded laterally, entoprocesses rounded, arcessus straight, apex as single lobe, hooked ventrally apically; gonosaccus well developed with uniform gonosetae present.

Female: unknown.

#### Etymology.

The species epithet refers to the diminutive size of this species.

#### Comments.

This new species of *Glenochrysa* is easily distinguished from other species in the genus by the relatively small size, reduced wing venation with few gradates, and limited markings and embossing on the wing membrane.

### Revised key to Australian species of *Glenochrysa* Esben-Petersen (after New 1980)

**Table:** 

1	Hind wing with conspicuous brown shading	***Glenochrysa franzeni Kimmins***
–	Hind wing unshaded	**2**
2	Forewing inner gradates almost transverse, aligned with dark transverse infuscate band	***Glenochrysa tillyardi* New**
–	Forewing inner gradates diagonal, sub-parallel to outer gradates	**3**
3	Both forewing and hind wing with two or fewer gradates in both inner and outer gradates series, gradate series poorly defined; 6 (rarely 7) r1-rs crossveins in either forewing or hind wing; forewing venation almost completely dark, wing membrane mostly hyaline except for shading along crossveins; north-western Australia	***Glenochrysa minima* sp. n.**
–	Both forewing and hind wing with three or fewer gradates in both inner and outer gradates series, gradate series poorly defined; 8 (rarely 7) r1-rs crossveins in either forewing or hind wing; forewing primary wing veins pale, crossveins darker, infuscate shading in wing membrane; north-eastern Australia	**4**
4	Frons and clypeus pale, with black genal mark, sometimes with black crescent like mark below antennal base	***Glenochrysa opposita* (McLachlan)**
–	Frons and clypeus more extensively marked with red	**5**
5	Red band across frons; large red mark on gena and onto clypeus	***Glenochrysa irregularis* (Banks)**
–	Narrow red band on gena stopping at lateral margin of clypeus, not crossing frons	***Glenochrysa regularis* (Banks)**

## Supplementary Material

XML Treatment for Glenochrysa minima
